# Resurgent sodium current promotes action potential firing in the avian auditory brainstem

**DOI:** 10.1113/JP275083

**Published:** 2018-01-04

**Authors:** Hui Hong, Ting Lu, Xiaoyu Wang, Yuan Wang, Jason Tait Sanchez

**Affiliations:** ^1^ Roxelyn and Richard Pepper Department of Communication Sciences and Disorders Northwestern University Evanston IL 60208 USA; ^2^ Department of Neurobiology Northwestern University Evanston IL 60208 USA; ^3^ The Hugh Knowles Hearing Research Center Northwestern University Evanston IL 60208 USA; ^4^ Department of Biomedical Sciences Florida State University Tallahassee FL 32306 USA; ^5^ Program in Neuroscience Florida State University College of Medicine Florida State University Tallahassee FL 32306 USA

**Keywords:** auditory system, development, sodium channel, nucleus magnocellularis, potassium channel, action potential, neuron

## Abstract

**Key points:**

- Auditory brainstem neurons of all vertebrates fire phase‐locked action potentials (APs) at high rates with remarkable fidelity, a process controlled by specialized anatomical and biophysical properties.
- This is especially true in the avian nucleus magnocellularis (NM) – the analogue of the mammalian anteroventral cochlear nucleus.
- In addition to high voltage‐activated potassium (K_HVA_) channels, we report, using whole cell physiology and modelling, that resurgent sodium current (*I*_NaR_) of sodium channels (Na_V_) is equally important and operates synergistically with K_HVA_ channels to enable rapid AP firing in NM.
- Anatomically, we detected strong Na_V_1.6 expression near hearing maturation, which was less distinct during hearing development despite functional evidence of *I*_NaR_, suggesting that multiple Na_V_ channel subtypes may contribute to *I*_NaR_.
- We conclude that *I*_NaR_ plays an important role in regulating rapid AP firing for NM neurons, a property that may be evolutionarily conserved for functions related to similar avian and mammalian hearing.

**Abstract:**

Auditory brainstem neurons are functionally primed to fire action potentials (APs) at markedly high rates in order to rapidly encode the acoustic information of sound. This specialization is critical for survival and the comprehension of behaviourally relevant communication functions, including sound localization and distinguishing speech from noise. Here, we investigated underlying ion channel mechanisms essential for high‐rate AP firing in neurons of the chicken nucleus magnocellularis (NM) – the avian analogue of bushy cells of the mammalian anteroventral cochlear nucleus. In addition to the established function of high voltage‐activated potassium channels, we found that resurgent sodium current (*I*_NaR_) plays a role in regulating rapid firing activity of late‐developing (embryonic (E) days 19–21) NM neurons. *I*_NaR_ of late‐developing NM neurons showed similar properties to mammalian neurons in that its unique mechanism of an ‘open channel block state’ facilitated the recovery and increased the availability of sodium (Na_V_) channels after depolarization. Using a computational model of NM neurons, we demonstrated that removal of *I*_NaR_ reduced high‐rate AP firing. We found weak *I*_NaR_ during a prehearing period (E11–12), which transformed to resemble late‐developing *I*_NaR_ properties around hearing onset (E14–16). Anatomically, we detected strong Na_V_1.6 expression near maturation, which became increasingly less distinct at hearing onset and prehearing periods, suggesting that multiple Na_V_ channel subtypes may contribute to *I*_NaR_ during development. We conclude that *I*_NaR_ plays an important role in regulating rapid AP firing for NM neurons, a property that may be evolutionarily conserved for functions related to similar avian and mammalian hearing.

## Key points

- Auditory brainstem neurons of all vertebrates fire phase‐locked action potentials (APs) at high rates with remarkable fidelity, a process controlled by specialized anatomical and biophysical properties.
- This is especially true in the avian nucleus magnocellularis (NM) – the analogue of the mammalian anteroventral cochlear nucleus.
- In addition to high voltage‐activated potassium (K_HVA_) channels, we report, using whole cell physiology and modelling, that resurgent sodium current (*I*_NaR_) of sodium channels (Na_V_) is equally important and operates synergistically with K_HVA_ channels to enable rapid AP firing in NM.
- Anatomically, we detected strong Na_V_1.6 expression near hearing maturation, which was less distinct during hearing development despite functional evidence of *I*_NaR_, suggesting that multiple Na_V_ channel subtypes may contribute to *I*_NaR_.
- We conclude that *I*_NaR_ plays an important role in regulating rapid AP firing for NM neurons, a property that may be evolutionarily conserved for functions related to similar avian and mammalian hearing.

## Introduction

Voltage‐dependent sodium (Na_V_) channels play a critical role in generation of action potentials (APs), the firing pattern of which is fundamental for information processing in the nervous system (Eijkelkamp *et al*. 2012). A unique property of some Na_V_ channels is a resurgent sodium current (*I*_NaR_) (Raman & Bean, 1997). *I*_NaR_ is the result of a voltage‐dependent open channel block of the Na_V_ α‐subunit by an intracellular particle that competes with the classic inactivation gate (i.e. the cytoplasmic linker between the III and IV domains of the α‐subunit) during depolarization. Channels that are in the ‘blocked’ state, but not the ‘classic‐inactivated’ state, generate *I*_NaR_ during AP repolarization because the open channel blocker loses affinity for the α‐subunit at repolarized membrane potentials. As a result, *I*_NaR_ provides a small depolarizing drive to the membrane near the AP threshold – a property that promotes repetitive neuronal firing. In addition, activation of the open channel blocker facilitates the recovery of Na_V_ channels after depolarization and increases Na_V_ channel availability by competing against classic inactivation (Raman & Bean, 2001). This specific blocker has been identified as the β4‐subunit in cerebellar Purkinje cells (Grieco *et al*. 2005; Aman *et al*. 2009), whereas multiple α‐subunits (e.g. Na_V_1.2, 1.5, 1.6 and 1.7) have been shown to be capable of carrying *I*_NaR_ (Rush *et al*. 2005; Jarecki *et al*. 2010). As a result of unique features of the open channel blocker, studies show that *I*_NaR_ plays an important role in promoting high rates of AP firing in numerous mammalian neurons, underlying their highly specialized information processing patterns (Lewis & Raman, 2014).

In the auditory brainstem of all vertebrates, neurons are known for their remarkable ability to fire APs at high rates (Oertel, 1997). This specialization is important for encoding sound in an ultrafast and temporally precise manner, properties essential for survival and comprehension of behaviourally relevant communication functions, including sound localization and signal extraction in complex listening environments (Shannon *et al*. 1995; Anderson *et al*. 2010; Grothe *et al*. 2010). An exemplar of this ability is well studied in the avian auditory brainstem. Neurons in nucleus magnocellularis (NM) – the avian analogue of bushy cells of the mammalian anteroventral cochlear nucleus – are able to phase‐lock to inputs up to 1000 Hz with high fidelity, as demonstrated by single‐unit recordings from barn owls (Koppl & Carr, 1997). Similarly, in the mammalian auditory brainstem, the calyx of Held is a pivotal synapse involved in the microsecond precision of sound localization computations. Here, fast Na_V_ channel kinetics (Leao *et al*. 2005) and abundant expression of high voltage‐activated potassium channels (K_HVA_) (Wang *et al*. 1998) along with *I*_NaR_ present at both pre‐ and postsynaptic sites (Leao *et al*. 2006; Kim *et al*. 2010) promote high rates of AP firing. Based on the aforementioned properties of *I*_NaR_ in mammals, as well as the established and shared functional phenotypes between species (Carr & Soares, 2002), we hypothesized that this unique current also plays an important role in shaping the fast firing pattern observed for avian NM neurons.

In this study we tested whether NM neurons present with *I*_NaR_. By investigating the function of *I*_NaR_ in AP firing rates of chicken NM neurons both experimentally and computationally, we found that NM neurons have robust *I*_NaR_ that significantly increases Na_V_ availability immediately after depolarization and facilitates Na_V_ channel recovery. Removal of *I*_NaR_ in a model NM neuron undermines its ability to fire at high rates. We also examined the maturation of *I*_NaR_ relative to hearing onset and the potential Na_V_ channel subtype that carries this current in developing NM using immunocytochemistry. We conclude that *I*_NaR_ plays an important role in regulating rapid AP firing for NM neurons, a property that may be evolutionarily conserved for functions related to similar avian and mammalian hearing.

## Methods

### *In vitro* electrophysiology in brainstem slices

#### Slice preparation

All animal procedures were approved by the Northwestern University and Florida State University Institutional Animal Care and Use Committees and conducted in accordance with the National Institutes of Health Guide for the Care and Use of Laboratory Animals. Acute brainstem slices were prepared from 96 White Leghorn chicken (*Gallus gallus domesticus*) embryos of either sex as previously described (Sanchez *et al*. 2011; Hong *et al*. 2016). Briefly, embryos were rapidly decapitated and the brain was dissected from the skull to isolate the brainstem region of interest. This procedure is consistent with the recommendation from the Panel on Euthanasia of the American Veterinary Medical Association and is appropriate for the species, stages of development and size of the embryos. For electrophysiological experiments, eggs were obtained from Sunnyside Farms, Inc. (Beaver Dam, WI, USA) and incubated in the central auditory physiology laboratory at Northwestern University. For immunocytochemical experiments, eggs were obtained from Charles River Laboratories (Wilmington, MA, USA) and incubated in a Florida State University vivarium. The authors understand and conform to the principles and regulations described by *The Journal of Physiology* (Grundy, 2015).

To study the stimulus frequency–firing pattern of NM neurons and the properties and function of *I*_NaR_, brainstem slices were taken from chickens at embryonic days (E) 19–21. Frequency‐firing pattern is defined as the calculated firing probability (see below) of APs as a function of stimulus frequency. At this age range, near‐mature hearing ability is established (Saunders *et al*. 1973; Rebillard & Rubel, 1981; Jones *et al*. 2006) and NM neurons have obtained mature‐like morphology and physiology (Jhaveri & Morest, 1982b). To study the development of *I*_NaR_, along with the development of frequency–firing pattern, chickens at the age of E11–12 and E14–16 were included in the current study, corresponding to before and during hearing onset, respectively, while chickens at E19–21 were considered as after hearing onset (Jones *et al*. 2006). The brainstem was dissected and isolated in ice‐cold (∼0°C) oxygenated low‐Ca^2+^, high‐Mg^2+^ modified artificial cerebral spinal fluid (ACSF) containing the following (in mm): 130 NaCl, 2.5 KCl, 1.25 NaH_2_PO_4_, 26 NaHCO_3_, 3 MgCl_2_, 1 CaCl_2_ and 10 glucose. ACSF was continuously bubbled with a mixture of 95% O_2_–5% CO_2_ (pH 7.4, osmolarity 295–310 mosmol l^−1^). The brainstem was blocked coronally, affixed to the stage of a vibrating blade microtome slicing chamber (Ted Pella, Inc., Redding, CA) and submerged in ice‐cold ACSF. Bilaterally symmetrical coronal slices were made (200–300 μm thick) and approximately three to seven slices (depending on age) containing NM were taken from caudal to rostral, roughly representing the low‐to‐high frequency regions, respectively. All neurons reported here were obtained from the rostral one‐half of the entire nucleus, roughly representing the mid‐ to high‐frequency regions of NM.

Slices were collected in a custom holding chamber and allowed to equilibrate for 1 h at room temperature in normal ACSF containing the following (in mm): 130 NaCl, 2.5 KCl, 1.25 NaH_2_PO_4_, 26 NaHCO_3_, 1 MgCl_2_, 3 CaCl_2_ and 10 glucose. Normal ACSF was continuously bubbled with a mixture of 95% O_2_–5% CO_2_ (pH 7.4, osmolarity 295–310 mosmol l^−1^). Slices were transferred to a recording chamber mounted on an Olympus BX51W1 (Center Valley, PA, USA) microscope for electrophysiological experiments. The microscope was equipped with a CCD camera, ×60 water‐immersion objective and infrared differential interference contrast optics. The recording chamber was superfused continuously with a motorized pump (Welco, Tokyo, Japan) at room temperature (monitored continuously with a bath emerged thermometer at ∼24°C, Warner Instruments, Hamden, CT, USA) in oxygenated normal ACSF at a rate of 1.5–2 ml min^−1^.

#### Whole cell electrophysiology

Voltage‐clamp and current‐clamp experiments were performed using an Axon Multiclamp 700B amplifier (Molecular Devices, Sunnyvale, CA, USA). Patch pipettes were pulled to a tip diameter of 1–2 μm using a P‐97 Flaming–Brown micropipette puller (Sutter Instrument, Novato, CA, USA) and had resistances ranging from 3 to 6 MΩ. For voltage‐clamp experiments of isolated Na_V_ currents, the internal solution was caesium‐based and contained the following (in mm): 150 CsCl, 10 NaCl, 0.2 EGTA and 10 HEPES, pH adjusted to 7.3–7.4 with CsOH. The Cs^+^‐based internal solution was used to block K_V_ currents and reduce space‐clamp issues. The junction potential was ∼−3 mV. Series resistance was compensated for by ∼80% in all voltage‐clamp recordings. For current‐clamp experiments, the internal solution was potassium‐based and contained the following (in mm): 105 potassium gluconate, 35 KCl, 1 MgCl_2_, 10 HEPES‐K, 5 EGTA, 4 4‐Mg_2_ATP, and 0.3 4‐Tris_2_GTP, pH adjusted to 7.3–7.4 with KOH. The junction potential was ∼−10 mV. Data in both voltage clamp and current clamp experiments were not corrected for junction potentials.

Pipettes were visually guided to NM and neurons were identified and distinguished from surrounding tissue based on cell morphology, known structure and location of the nucleus within the slice. After a gigaohm seal was attained, membrane patches were ruptured and neurons were first held in the voltage clamp mode of the whole‐cell configuration. A small hyperpolarizing (−1 mV, 30 ms) voltage command was presented to monitor whole‐cell parameters (i.e. cell membrane capacitance, series resistance and input resistance). NM neurons were included in the data analysis only if they had series resistances <15 MΩ. Raw data were low‐pass filtered at 5 kHz and digitized at 50 kHz using a Digidata 1440A (Molecular Devices).

All experiments were conducted in the presence of the GABA_A_ receptor antagonist picrotoxin (100 μm). Synaptic glutamate transmission was continuously blocked using dl‐2‐amino‐5‐phosphonopentanoic acid (dl‐APV, 100 μm, an NMDA receptor antagonist) and 6‐cyano‐7‐nitroquinoxaline‐2,3‐dione (CNQX, 20 μm, an AMPA receptor antagonist). In current‐clamp experiments, tetraethylammonium (TEA, 1 mm) was used to block K_V_3‐containing channels. Current commands of square pulse trains (duration 1 s) were injected into the soma of E19–21 NM neurons before and during TEA application. The current strength was 1 nA and individual square pulse width was 2 ms. Square pulse trains were applied at varying frequencies: 50, 100, 150, 200, 250 and 300 Hz. In order to profile the frequency–firing pattern for NM neurons, firing probability per square pulse (for simplicity, ‘firing probability’) was calculated as the number of APs divided by the total number of square pulses and plotted as a function of stimulus frequency. To study the development of frequency–firing pattern, the same current commands of square pulse trains were used for NM neurons at E14–16. For NM neurons at E11–12, however, individual square pulse width was extended to 5 ms, because neurons at this age are presented with significantly wider AP half‐width (average = 4.6 ms) than the other two age groups (Hong *et al*. 2016). In addition, square pulse trains were applied at different frequencies: 10, 30, 50, 70, 100, 120 and 150 Hz.

In voltage‐clamp experiments, isolated Na_V_ currents were recorded with bath application of TEA (3 mm), 4‐AP (30 μm) and CdCl_2_ (0.2 mm) to block potassium and calcium channels. Classic voltage‐clamp protocols to elicit *I*_NaR_ were used for NM neurons (Raman & Bean, 1997; see Results). Briefly, repolarizations at membrane voltages from −70 to 0 mV (in a step of 5 mV) were applied after a depolarizing conditioning step. Three levels of conditioning step were used, +30, 0 and −30 mV, along with two durations, 10 and 100 ms. *I*_NaR_ properties were characterized separately under these six experimental conditions. Afterwards, tetrodotoxin (TTX, 1 μm) was bath applied and the same voltage‐clamp protocols were repeated during TTX application. Data reported in this study were obtained by subtracting the TTX‐insensitive current traces from the control traces. Capacitive currents generated during voltage‐clamp recordings were blanked or reduced offline.

#### Data analysis

Recording protocols were written and run using Clampex acquisition and Clampfit analysis software (v. 10.3; Molecular Devices). Statistical analyses and graphing protocols were performed using Prism (v. 7.0b; GraphPad Software, La Jolla, CA, USA) and MATLAB (v. R2014b; The MathWorks, Natick, MA, USA) software. Student's *t* test or analysis of variance (ANOVA) with *post hoc* Bonferroni adjusted *t* test was used to determine significance. The standard for significant differences was defined as *P* < 0.05. Numerical values in the text are reported as means ± standard deviation (SD). Numerical values in Table 3 are reported as means ± standard error of the mean (SEM). Error bars in all figures represent SEM.

#### Reagents

All bath‐applied drugs except for TTX were allowed to perfuse through the recording chamber for ∼10 min before subsequent recordings. TTX application was allowed for 3–5 min before subsequent recordings. dl‐APV, CNQX and all other salts and chemicals were obtained from Sigma‐Aldrich (St Louis, MO, USA). Picrotoxin was obtained from Tocris Bioscience (Ellisville, MO, USA). TTX was obtained from Alomone Labs (Jerusalem, Israel). TEA was obtained from VWR (Radnor, PA, USA).

### Computational modelling

**Figure 1:**
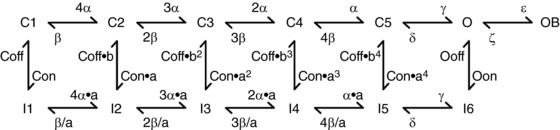
Markovian state model of the sodium (Na_V_) channel Reproduced from the model described by Khaliq *et al*. (2003). C, I, O, and OB denote closed, inactivated, open and open‐blocked states, respectively. The values of the kinetic parameters are shown in Table 2.

Based on NM models previously described in detail elsewhere (Howard & Rubel, 2010; Lu *et al*. 2017), a single‐compartment computational model was constructed using NEURON 7.1 (Table 1) (Hines & Carnevale, 1997). This model contains currents mediated by low and high voltage‐activated potassium channels (K_LVA_ and K_HVA_, respectively), Na_V_ and passive leak channels. Modelling schemes were identical to those described in Lu *et al*. (2017) for all membrane currents except for Na_V_ currents (Table 2). The Hodgkin–Huxley style formalism, previously employed to model Na_V_ current, was replaced with a Markovian 13‐state Na_V_‐channel model, which generates the transient (*I*_NaT_), persistent (*I*_NaP_) and resurgent (*I*_NaR_) current components simultaneously (Fig. 1) (Khaliq *et al*. 2003). We adopted a modified version described previously (Akemann & Knopfel, 2006), which includes the *Q*_10_ parameter to adjust the Na_V_ current to match the experimental recording temperature of 24°C. *Q*_10_ is a measure of the degree to which a biological process depends on temperature. It is defined as the ratio between the rate of a biological process at two temperatures separated by 10°C. In the context of ion channels, it can be applied to the temperature dependence of the rate of channel opening and closing and to the dependence of maximum channel conductance on temperature.

**Table 1:** Single compartment model

Parameter	Value
Axial resistance	50 Ω cm
Temperature	24°C
*E*_Na_	44 mV
*E*_K_	−80 mV
Length	20 μm
Diameter	20 μm
*g*_Leak_	0.002 S cm^−2^
*g*_NaV_	0.025 S cm^−2^
*g*_KLVA_	0.0069 S cm^−2^
*g*_KHVA_	0.004 S cm^−2^

**Table 2:** Model parameters

Parameter	Value
*I*_NaV_, Markovian model	
α	150exp(*V*/20) ms^−1^
β	3exp(−*V*/20) ms^−1^
γ	150 ms^−1^
δ	40 ms^−1^
ε	1.75 ms^−1^
ζ	0.03exp(−*V*/25) ms^−1^
*C*_on_	0.005 ms^−1^
*C*_off_	0.5 ms^−1^
*O*_on_	0.75 ms^−1^
*O*_off_	0.005 ms^−1^
*a*	(*O*_on_/*C*_on_)^1/4^
*b*	(*O*_off_/*C*_off_) ^1/4^
*Q*_10_	3
*T*_0_	22°C
*I*_KLVA_, Hodgkin–Huxley model	
*w_∞_*	1/(1 + exp(−(*V* + 67)/8)
*z_∞_*	1/(1 + exp(−(*V* + 71)/10)
τ_w_	(100/(6 × exp((*V* + 60)/6) + 16 × exp(−(*V* + 60)/45)) + 1.5
τ_z_	(100/(exp((*V* + 60)/20) + exp(−(*V* + 60)/8)) + 50
*Q*_10_	3
*T*_0_	22°C
*I*_KHVA_, Hodgkin–Huxley model	
*n_∞_*	1/(1 + exp(−(*V* + 42.5)/4.5)
*p_∞_*	1/(1 + exp(−(*V* + 11.2)/6)
τ_n_	(100/(11 × exp((*V* + 60)/24) + 21 × exp(−(*V* + 60)/23)) + 0.7
τ_p_	(100/(4 × exp((*V* + 60)/32) + 5 × exp(−(*V* + 60)/22)) + 5
*Q*_10_	3
*T*_0_	22°C

*V*, *C* and *O* denote voltage, closed and opened states, respectively.

To remove the *I*_NaR_ component, the rate constant for the O→OB transition, ε, was set to zero. This resulted in considerable slowing of *I*_NaT_ decay (see Fig. 6 *B*, blue trace). To modify *I*_NaR_ without affecting *I*_NaT_ and *I*_NaP_, we applied the method described previously (Magistretti *et al*. 2006). Kinetic constants were modified as follows: ε was set to 0; *O*_on_ and *O*_off_ were increased to 2.15 ms^−1^ and 0.01433 ms^−1^ to restore *I*_NaT_ and *I*_NaP_, respectively (see Fig. 6 *B* and *C*).

### Immunocytochemistry

Chicken embryo brains (E11, E15 and E21; *n* = 3 for each age) were dissected out of the skull and immediately immersed in modified periodate–lysin–paraformaldehyde (PLP) fixative: 0.2% (w/v) paraformaldehyde, 2.7% (w/v) lysin HCl, 0.21% (w/v) NaIO_4_, and 0.1% (w/v) Na_2_HPO_4_ (Kuba *et al*. 2005). Brains of all ages were then transferred to 30% sucrose in 0.1 m phosphate buffer for 3 days and sectioned in the coronal plane at 30 μm on a freezing sliding microtome. Each section was collected in 0.01 m phosphate‐buffered saline (PBS) with 0.02% sodium azide. Alternate serial sections were immunocytochemically stained for anti‐Na_V_1.6, generously provided by Dr Hiroshi Kuba at Kyoto University (Kuba *et al*. 2006). Briefly, free‐floating sections were incubated with primary antibody solutions (1:1000) diluted in PBS with 0.3% Triton X‐100 overnight at 4°C, followed by Alexa‐Fluor secondary antibodies (Thermo Fisher Scientific, Waltham, MA, USA) at 1:400 overnight at 4°C. Sections from E21 chickens were double stained with Neurofilament 200 (Sigma‐Aldrich, N0142). Sections of all ages were then mounted on gelatin‐coated slides and coverslipped with Fluoromount‐G mounting medium^®^ (Southern Biotech, Birmingham, AL, USA). Images were captured with an Olympus FV1200 confocal microscope. Image brightness, gamma and contrast adjustments were performed in Adobe Photoshop (Adobe Systems, San Jose, CA, USA).

## Results

### Frequency–firing pattern of NM neurons

**Figure 2:**
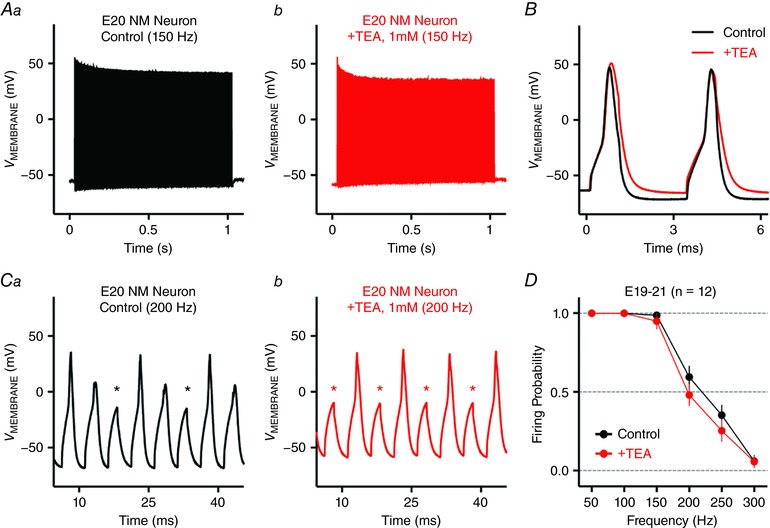
Frequency–firing pattern of NM neurons at E19–21 *Aa* and *b*, representative voltage responses recorded from an E20 NM neuron to current injections of square pulse trains at 150 Hz, before and during TEA (1 mm) application, respectively. *B*, overlaid enlargement of representative voltage responses shown in *Aa* and *b*. *Ca* and *b*, representative voltage responses to current injections of square pulse trains at 200 Hz, before and during TEA application, respectively. Asterisk indicates action potential failure. *D*, population data showing firing probability of NM neurons as a function of square pulse frequency. The strength of all square pulse trains is 1 nA with a duration of 1 s. Individual square pulse width is 2 ms. Error bar, SEM.

Auditory brainstem neurons are able to fire APs at high rates (Oertel, 1997; Trussell, 1997, 1999). Therefore, we first tested whether late‐developing NM neurons (E19–21) follow varying frequency rates with high fidelity. Square pulse current trains at frequencies from 50 to 300 Hz were injected into the soma of NM neurons. Firing probability at each frequency was defined as the average number of APs per square pulse (see Methods). The majority of NM neurons (11 out of 12 neurons) were able to follow square pulse trains of 150 Hz in a one‐to‐one manner (i.e. high‐fidelity, Fig. 2 *Aa* and *D*). Firing probability dropped to ∼0.6 at 200 Hz when obvious failures of spike generation are observed during the stimulus (Fig. 2 *Ca* and *D*, asterisks). When increasing the stimulus frequency beyond 200 Hz, firing probability was further reduced below 0.5 and in response to 300 Hz stimulation, NM neurons only fired a single onset AP (data not shown). Therefore, NM neurons are capable of firing APs at rates up to 200 Hz with good fidelity, and this ability of frequency firing is similar to other auditory brainstem neurons (Gao & Lu, 2008).

Our current‐clamp recordings were conducted at room temperature (∼24°C). Chickens have a body temperature of ∼42°C. Increasing recording temperature changes the physiology of neurons dramatically (Kushmerick *et al*. 2006). Our previous study recorded APs at near‐physiological temperature (∼35°C) and we showed a significant improvement of AP kinetics for NM neurons (Hong *et al*. 2016). For example, AP half‐width was reduced by ∼30% and fall rate increased by ∼40% when increasing the temperature to ∼35°C. Based on these observations, one would expect that NM neurons recorded at higher temperatures could follow stimulations higher than 200 Hz. Indeed, a recent study using current‐clamp recordings at 38–40°C reveals that NM neurons are able to follow square pulse trains up to 500 Hz with good fidelity, whereas the firing probability drops dramatically at 667 Hz (Kuba *et al*. 2015).

K_V_3‐containing channels, which belong to the family of K_HVA_ channels, promote the AP repolarizing phase and have been shown to shape the frequency–firing pattern of many neurons (Wang & Kaczmarek, 1998; Johnston *et al*. 2010; Hong *et al*. 2016). Based on previous findings, we hypothesized that K_v_3‐containing channels play a major role in regulating the rapid firing capability NM neurons. To test this hypothesis, we bath applied TEA (1 mm) to block K_V_3‐containing channels. Representative responses to 150 Hz square pulse trains before and during TEA application are shown in Fig. 2 *Aa* and *b*. The enlargement of two overlaid APs from the same neuron is shown in Fig. 2 *B*. During TEA application, the majority of NM neurons (10 out of 12 neurons) were still able to follow pulse trains of 150 Hz with 100% fidelity, despite a significant widening of APs (*P* = 0.0034, Fig. 2 *B* and *D*). Correspondingly, no changes in firing probability at 50 or 100 Hz were observed with blockade of K_V_3‐containing channels (Fig. 2 *D*). In contrast, we observed reduction in firing probability when increasing the stimulus frequency to 200 or 250 Hz (Fig. 2 *Ca* and *b* and *D*). In response to 200 Hz stimulation, firing probability was reduced by ∼19% on average. Although this reduction is statistically significant (*P* = 0.0011), nearly half of the recorded neurons (5 out of 12 neurons) showed a reduction of only ∼10% or less in firing probability. These observations with TEA application indicate that K_V_3‐containing channels contribute partially to higher frequency firing of NM neurons and thus gave rise to an important question: what other factor(s) regulate the ability of NM neurons to follow inputs up to 200 Hz with relatively high fidelity?

### Resurgent Na_V_ current of NM neurons

**Figure 3:**
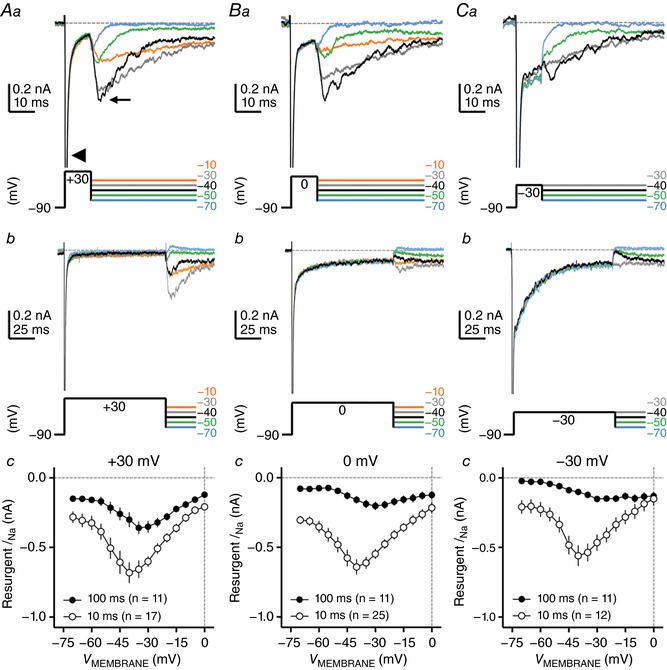
Resurgent sodium current (*I*_NaR_) of NM neurons at E19–21 *Aa* and *b*, *Ba* and *b* and *Ca* and *b*, representative current traces in response to voltage‐clamp protocols that elicit *I*_NaR_ shown below traces. The amplitude of the conditioning step is +30 mV in *Aa* and *b*, 0 mV in *Ba* and *b* and −30 mV in *Ca* and *b*. The duration of the conditioning step is 10 ms in *Aa–Ca* and 100 ms in *Ab–Cb*. Arrowhead and small arrow in *Aa* indicate the transient sodium current (*I*_NaT_) and *I*_NaR_, respectively. *Ac*, *Bc* and *Cc*, population data showing the *I*_NaR_ amplitude as a function of repolarizing membrane voltage (*V*_MEMBRANE_) in response to the conditioning steps shown in *Aa* and *b*, *Ba* and *b* and *Ca* and *b*, respectively. Error bar, SEM.

A number of previous studies suggest that *I*_NaR_ shapes the rapid firing and burst generation in many mammalian neurons (Lewis & Raman, 2014). Therefore, we speculated that NM neurons have *I*_NaR_, which is likely one of the factors that regulate the frequency–firing pattern of NM neurons. We first examined whether *I*_NaR_ is present in NM by using classic voltage‐clamp protocols (Raman & Bean, 1997; Fig. 3). NM neurons were held at −90 mV before giving a depolarizing conditioning step to +30 mV (duration = 10 ms). A transient Na_V_ current (*I*_NaT_) was evoked by the conditioning step (Fig. 3 *Aa*, arrowhead). Afterwards, neurons were repolarized to varying membrane voltages from −70 mV to 0 mV (Δ step = 5 mV). Inward‐going *I*_NaR_, which could be eliminated by TTX application, was elicited by repolarization and its amplitude was plotted as a function of repolarizing membrane voltage (Fig. 3 *Aa*, small arrow, and *Ac*). The *I–V* curve of *I*_NaR_ for NM neurons peaked at −40 mV and presented with the typical ‘V’ shape that largely resembled that of *I*_NaR_ reported in mammalian neurons (Lewis & Raman, 2014). The maximal amplitude of *I*_NaR_ is usually less than 1 nA for NM neurons recorded from slice preparation, which is much smaller than the amplitude of *I*_NaT_ (Fig. 3 *Ac*). This property is also similar to that observed in other auditory brainstem neurons (Leao *et al*. 2006; Kim *et al*. 2010). Therefore, by using the conditioning step of +30 mV at 10 ms, NM neurons show robust generation of *I*_NaR_ that is present with similar amplitude and voltage dependence properties to mammalian neurons.

In mammalian neurons, the *I*_NaR_ amplitude is dependent on the duration and level of the conditioning step (Raman & Bean, 2001). This is due to two competing inactivation mechanisms of Na_V_ channels: open channel block induced by an intracellular particle and the classic inactivation induced by the ‘gate’ (i.e. cytoplasmic III–IV linker) within the α‐subunit. As demonstrated in cerebellar Purkinje cells, the majority of Na_V_ channels will be open channel blocked if neurons are exposed to a short and more positive depolarization (e.g. an AP). The unbinding of open channel block at moderately negative membrane voltage (e.g. −40 mV) under this condition generates large *I*_NaR_. With longer and less positive depolarization, Na_V_ channels will mainly adopt the classic inactivation gate that requires hyperpolarization of membrane voltage to be released, and the generation of *I*_NaR_ will be minimal. In order to investigate whether *I*_NaR_ in NM has similar properties, we applied conditioning steps with different durations and levels and compared the *I*_NaR_ amplitude among the conditions. For example, the duration of +30 mV depolarization was extended from 10 to 100 ms. Fig. 3 *Ab* shows representative current traces in response to the prolonged conditioning step. The generation of *I*_NaR_ can still be observed but with a smaller amplitude. Indeed, comparison between the two *I–V* curves revealed a significant reduction in current amplitude when the conditioning step was elongated to 100 ms (Fig. 3 *Ac*). Interestingly, we also observed a shift in peak membrane voltage towards the positive direction by ∼5 mV.

Next, we changed the level of the conditioning step to 0 mV and −30 mV while maintaining the same duration (i.e. 10 ms). Fig. 3 *Ba* and *Ca* shows representative current traces in response to conditioning steps of 0 mV and −30 mV, respectively. By comparing the *I–V* curves shown in Fig. 3 *Ac*–*Cc*, we observed a decreasing trend (albeit not significant) in *I*_NaR_ amplitude when the conditioning step was switched from positive to negative voltages. The voltage dependence did not change with different levels of conditioning steps (i.e. all peaked at −40 mV). Finally, we applied a long duration (i.e. 100 ms) conditioning step at non‐positive levels (i.e. 0 and −30 mV), with the prediction that Na_V_ channels will be unlikely to adopt the open channel block and thus elicit minimal *I*_NaR_. Indeed, a dramatic reduction in current amplitude was observed in both cases (Fig. 3 *Bb* and *c* and *Cb* and *c*) along with a shift in voltage dependence by ∼10 mV. Under the condition of −30 mV, the reduction was so great that the typical ‘V’ shape of the *I*_NaR_ *I–V* curve was lost (Fig. 3 *Cc*), which can be explained by the following two factors. First, *I*_NaR_, if any, was hardly detectable in response to the conditioning step of −30 mV at 100 ms (Fig. 3 *Cb*). Instead, steady‐state current was evident following the repolarization. Second, low‐level contaminant noise and/or other channel conductances can obscure small *I*_NaR_, which prevents the amplitude of *I*_NaR_ from being reliably measured (Afshari *et al*. 2004; Aman *et al*. 2009).

**Figure 4:**
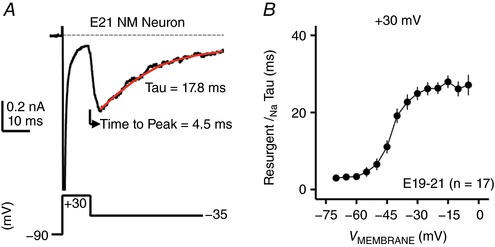
*I*_NaR_ kinetics of NM neurons at E19–21 *A*, representative current trace showing the calculation of time to peak and decay time constant (tau) for *I*_NaR_. Decay time constant (tau) is obtained by fitting a single exponential (red trace) to the decay phase of *I*_NaR_. The conditioning step is +30 mV at 10 ms. *B*, population data showing the decay time constant (tau) of *I*_NaR_ as a function of repolarizing membrane voltage (*V*_MEMBRANE_). Error bar, SEM.

We further characterized the kinetics of *I*_NaR_, which is considered to be another important property. Two variables of kinetics were used based on previous studies: time to peak and decay time constant (tau; Raman & Bean, 1997; Lewis & Raman, 2011). Fig. 4 *A* shows the calculation of two variables in the current study. Time to peak is defined as the time interval between the onset of repolarization and the *I*_NaR_ peak. Decay time constant was calculated by fitting a single exponential to the decay phase of *I*_NaR_. All calculations were conducted under the conditioning step of +30 mV at 10 ms. Across the population of NM neurons, the average time to peak was 3.97 ± 1.19 ms when measured at the membrane voltage that elicited the maximum current. The membrane voltage for the representative trace shown in Fig. 4 *A* was −35 mV. The decay time constant was plotted as a function of repolarizing membrane voltage (Fig. 4 *B*). Again, similar to mammalian neurons, the decay time constant of *I*_NaR_ increased with more depolarized membrane voltage and was generally larger than the decay variable of *I*_NaT_ (Ming & Wang, 2003; Rush *et al*. 2005), which confirmed the slower kinetics of *I*_NaR_. In summary, these observations suggest that the use of the open channel blocker, the inactivation mechanisms and the *I*_NaR_ kinetics of NM neurons closely resemble those of mammalian neurons, properties likely conserved across various species and neural structures.

### Function of resurgent Na_V_ current for NM neurons: experimental results

**Figure 5:**
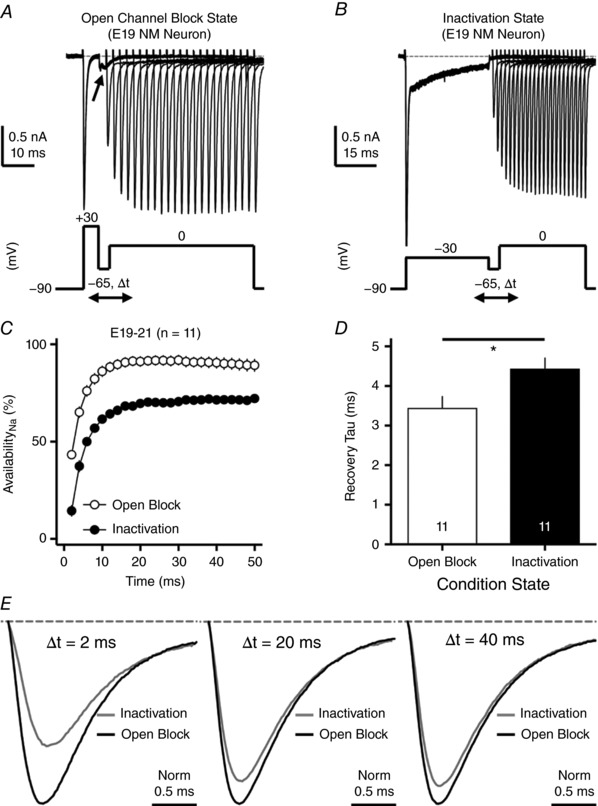
*I*_NaR_ helps increase Na_V_ channel availability and facilitates Na_V_ recovery *A* and *B*, representative current traces in response to voltage‐clamp protocols shown below traces. The conditioning step is +30 mV at 5 ms in *A* (open channel block state) and −30 mV at 40 ms in *B* (inactivation state). Δ*t* represents the varying recovery time, increasing from 2 to 50 ms in steps of 2 ms. Arrow in *A* indicates the generation of *I*_NaR_. *C*, population data showing the Na_V_ channel availability (%) as a function of recovery time. In order to calculate Na_V_ channel availability, a reference pulse to 0 mV was applied to NM neurons (not shown in the figure), and the amplitude of *I*_NaT_ after the recovery was normalized to this ‘reference amplitude’. Before the normalization, the amplitude of *I*_NaT_ was first adjusted by subtracting the steady‐state current that remained at the end of the conditioning step. The recovery trajectory is fit by a single exponential, in order to obtain recovery time constant (tau) shown in *D*. *D*, population data showing the recovery time constant (tau) under two different condition states. ^*^ *P* < 0.05. Numbers on bars represent sample size. *E*, representative current traces shown in *A* and *B* are normalized and overlaid for recovery time periods of 2 ms (left), 20 ms (middle) and 40 ms (right). Error bar, SEM.

We hypothesized that *I*_NaR_, along with the underlying open channel blocker, plays an important role in promoting the AP firing rates of NM neurons. We predicted that a neuron's activation of open channel blocker would promote Na_V_ channel availability and recovery from depolarization, which is ultimately important for subsequent and rapid AP firing. Therefore, we applied two voltage‐clamp protocols based on previous studies to test our prediction (Raman & Bean, 2001; Patel *et al*. 2015). In the first protocol, NM neurons were held at −90 mV before being depolarized to +30 mV for 5 ms (Fig. 5). According to our previous observations (see Fig. 3), this conditioned the majority of Na_V_ channels to be occupied by the open channel blocker (referred here as the ‘open channel block state’). The membrane voltage was then set at −65 mV for NM neurons to recover. The recovery time varied from 2 to 50 ms (Δ step = 2 ms). Finally, a depolarization to 0 mV was applied to evoke an *I*_NaT_. In the second protocol, the conditioning step was changed to −30 mV for 40 ms, in order to maximize the occupancy of the classic inactivation gate (referred to here as the ‘inactivation state’). Fig. 5 *A* and *B* shows representative current traces in response to the two protocols from the same neuron. When neurons were given a short and positive conditioning step, abrupt repolarization to the resting state resulted in an obvious generation of *I*_NaR_ (arrow in Fig. 5 *A*), while this was not observed when conditioned to the inactivation state (Fig. 5 *B*). The generation of *I*_NaR_ further supported the expectation that the occupancy of the open channel blocker was primary for the first protocol.

To determine Na_V_ channel availability under the two protocols, we calculated the normalized ratio, which indicates the amount of available Na_V_ channels after the recovery period. To do this, a reference pulse to 0 mV was applied to NM neurons prior to the implementation of the two protocols described above, and the amplitude of *I*_NaT_ after the recovery was normalized to this ‘reference amplitude’. The normalized ratio was plotted as a function of the recovery time in Fig. 5 *C*. We observed a clear separation of recovery trajectories between the two conditions, which indicates that the presence of open channel block significantly increased Na_V_ channel availability during the recovery. The recovery trajectory was fit with a single exponential in order to obtain a recovery time constant (tau). The open channel block significantly shortened the recovery time constant, facilitating the recovery of Na_V_ channels (Fig. 5 *D*). These observations provide supporting evidence for our prediction that the use of the open channel blocker can promote the availability and recovery of Na_V_ channels.

In addition, we found that the distinction between the two states was larger when the amount of recovery time was short. For example, when NM neurons were only given 2 ms to recover, the availability of Na_V_ channels was reduced by ∼67% on average from the open channel block state to the inactivation state (Fig. 5 *E*, left). But this difference reduced to ∼20% when the recovery time was longer than 20 ms (Fig. 5 *E*, middle and right). This suggests that the role of open channel block in recovering Na_V_ channels is more critical when the recovery time is limited, which is reminiscent of a highly restricted interspike interval when NM neurons are performing rapid auditory tasks (Warchol & Dallos, 1990; Jones & Jones, 2000).

### Function of resurgent Na_V_ current for NM neurons: computational results

**Figure 6:**
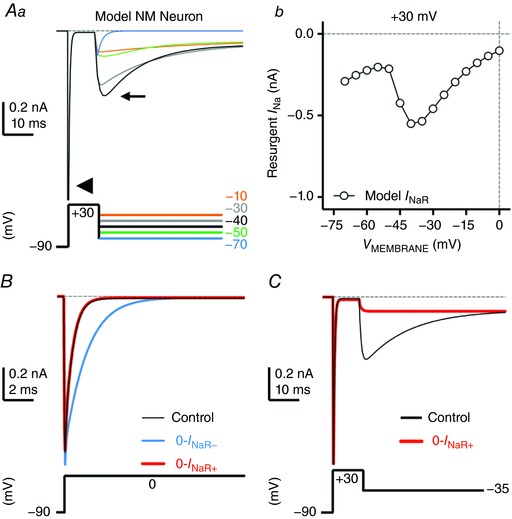
Simulations of Na_V_ currents from the model NM neuron *Aa*, simulated current traces (upper panel) in response to the voltage‐clamp protocol (lower panel) consisting of a 10 ms conditioning step to +30 mV, followed by step repolarizations to −10, −30, −40, −50 and −70 mV. Arrowhead and small arrow indicate *I*_NaT_ and *I*_NaR_, respectively. *Ab*, current–voltage relationship of simulated *I*_NaR_. *B*, simulated *I*_NaT_ (upper panel) was evoked by depolarizing steps to 0 mV (lower panel). The current obtained under control condition is shown in black. Switching off *I*_NaR_ by setting the rate constant for the O→OB transition, ε, to 0 resulted in considerable slowing of *I*_NaT_ decay (0‐*I*_NaR−_ condition, blue trace). *I*_NaT_ was restored under 0‐*I*_NaR+_ condition (ε = 0, *O*_on_ = 2.15 ms^−1^, and *O*_off_ = 0.01433 ms^−1^; red trace). *C*, simulated *I*_NaR_ under control (black trace) and 0‐*I*_NaR+_ condition (red trace). Removal of *I*_NaR_ under the 0‐*I*_NaR+_ condition has no effect on persistent Na_V_ current (*I*_NaP_).

We further used a computational model to examine the function of *I*_NaR_ in NM. This model is designed based on our previous study (Lu *et al*. 2017), in combination with the *I*_NaR_ model from Khaliq *et al*. (2003). The model NM neuron was held at −90 mV before giving a depolarizing conditioning step to +30 mV (duration = 10 ms). An *I*_NaT_ was evoked by the conditioning step (Fig. 6 *Aa*, arrowhead). The model NM neuron was then repolarized to varying membrane voltages from −70 mV to 0 mV (Δ step = 5 mV). The model NM neuron displayed slightly smaller *I*_NaR_ amplitudes (Fig. 6 *Aa*, small arrow) but comparable voltage dependence properties to our experimental data (Figs 6 *Ab* and 3 *Ac*). In order to remove *I*_NaR_, we first set the rate constant ε to zero (see Fig. 1 for reference). This modification, however, significantly slowed down the falling phase of *I*_NaT_ (Fig. 6 *B*, the ‘0‐*I*_NaR−_’ condition, blue trace). The slower falling phase of *I*_NaT_ is because the Markovian 13‐state Na_V_‐channel model sets the O→OB transition (with the rate constant ε, see Fig. 1) as a major exit path from the open state (Magistretti *et al*. 2006). Removing this path resulted in the slower speed of channels exiting the open state and thus led to a slower *I*_NaT_ falling phase. Therefore, we next increased the rate constant *O*_on_ to 2.15 ms^−1^ and the *O*_off_ to 0.01433 ms^−1^ to restore the normal decay kinetics of *I*_NaT_ and amplitude of *I*_NaP_, respectively (Fig. 6 *B* and *C*, the ‘0‐*I*_NaR+_’ condition, red trace). After these two modifications, *I*_NaR_ was successfully eliminated (Fig. 6 *C*). Both 0‐*I*_NaR−_ (blue) and 0‐*I*_NaR+_ (red) conditions were used to characterize the spiking activity of model NM neuron without *I*_NaR_. The only difference between 0‐*I*_NaR+_ and control conditions was the absence of *I*_NaR_, while the 0‐*I*_NaR−_ condition showed both the absence of *I*_NaR_ and slower *I*_NaT_ decay kinetics.

**Figure 7:**
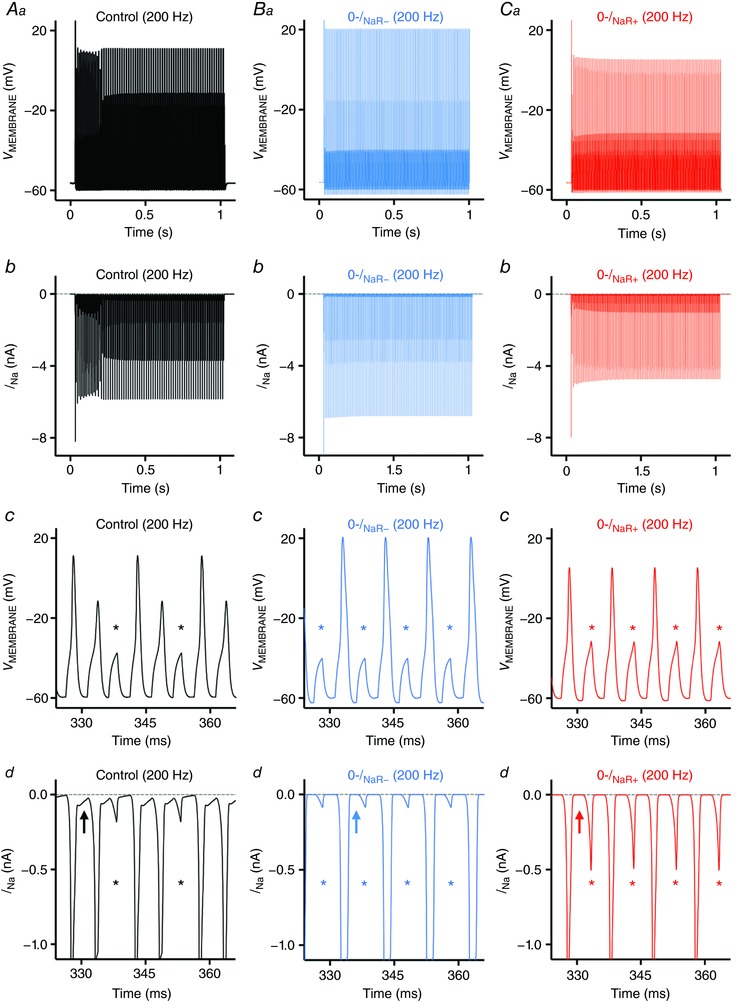
*I*_NaR_ promotes frequency firing of model NM neuron *Aa*–*d*, *Ba*–*d* and *Ca*–*d*, simulated voltage and current responses to square pulse current injections of 200 Hz, under the control, 0‐*I*_NaR−_ (i.e. *I*_NaR_ removal and slower *I*_NaT_) and 0‐*I*_NaR+_ (i.e. *I*_NaR_ removal) conditions, respectively. *Aa*, *Ba* and *Ca* are model output of membrane voltage (*V*_MEMBRANE_); enlarged traces shown in *Ac*, *Bc* and *Cc*. *Ab*, *Bb* and *Cb* are model output of Na_V_ currents; enlarged traces are shown in *Ad*, *Bd* and *Cd*. Asterisk indicates action potential failure. Arrows indicate the generation (*Ad*) or elimination (*Bd* and *Cd*) of *I*_NaR_.

Our model NM neuron generated similar voltage responses to square pulse current trains with varying frequencies, as compared to our experimental data. For example, the model NM neuron responded to a 200 Hz current injection with ∼0.6 AP firing probability (Fig. 7 *Aa* and *c*). It should be noted that an AP was identified only if the evoked voltage peak exceeded −30 mV. Voltages below this value were considered as failures (Fig. 7 *Ac*, asterisks). Fig. 7 *Ab* shows the total underlying Na_V_ current required for AP generation in Fig. 7 *Aa*. With the current and time scale expanded, we observed a clear but small inward Na_V_ current immediately after a large *I*_NaT_ (Fig. 7 *Ad*, arrow). This inward Na_V_ current is reminiscent of *I*_NaR_ that occurs during the AP repolarizing phase and closely resembles the data reported by previous ‘AP‐clamp’ studies in mammalian neurons (Raman & Bean, 1997, 1999). When we removed *I*_NaR_ in the model NM neuron, the reduction in firing probability was identical in both 0‐*I*_NaR−_ and 0‐*I*_NaR+_ conditions. Firing probability at 200 Hz was reduced by ∼21% (Fig. 7 *Ba*–*c*, asterisks; Fig. 7 *Ca*–*c*, asterisks) and the small inward Na_V_ current after *I*_NaT_ was no longer visible (Fig. 7 *Bd* and *Cd*, arrow). This result further confirmed that the small inward current is *I*_NaR_ and is induced by the mechanism of open channel block. In addition, when we removed both *I*_NaR_ and K_HVA_ currents, firing probability in response to 200 Hz stimulation was reduced by ∼23% from the control – just slightly larger than the reduction we observed with *I*_NaR_ removal only (data not shown).

It should be noted that *I*_NaT_ generated in the 0‐*I*_NaR−_ condition was obviously wider than in the 0‐*I*_NaR+_ condition, though the same amount of reduction in firing probability was observed for both conditions (Fig. 7 *Bd* and *Cd*). This difference is due to the slower decay kinetics of *I*_NaT_ in the 0‐*I*_NaR−_ condition that means Na_V_ channels inactivate more slowly (see Fig. 6 *B*). As a result, APs generated in the 0‐*I*_NaR−_ condition showed longer half‐width and larger amplitude than in the 0‐*I*_NaR+_ condition (Fig. 7 *Bc* and *Cc*). In addition, we also observed a difference between two conditions in a small *I*_NaT_ that failed to generate an AP after a large *I*_NaT_ (Fig. 7 *Bd* and *Cd*, asterisks). In the 0‐*I*_NaR−_ condition, Na_V_ currents after a large *I*_NaT_, the amplitude of which indicates the number of Na_V_ channels recovered after an AP, were present with smaller amplitude than those in the 0‐*I*_NaR+_ condition (Fig. 7 *Bd* and *Cd*, asterisks). This is because of the slower Na_V_ channel kinetics in the 0‐*I*_NaR−_ condition and the highly restricted recovery time in response to 200 Hz square pulse trains. Nevertheless, the identical reduction in firing probability in both conditions confirmed that the reduced ability of the model NM neuron to follow inputs at high rates was not due to a slower *I*_NaT_ but to the absence of *I*_NaR_. Together, our modelling results indicate that *I*_NaR_ helps shape AP firing rate of NM neurons.

### Development of resurgent Na_V_ current in NM

**Figure 8:**
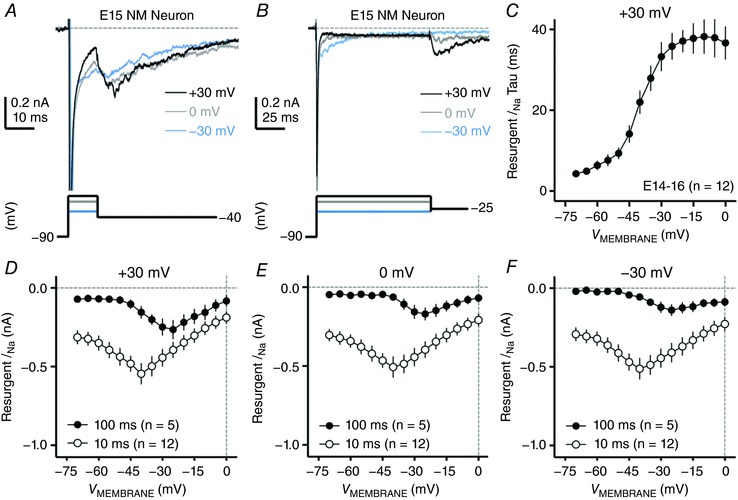
*I*_NaR_ properties of NM neurons at E14–16 *A* and *B*, representative current traces in response to voltage‐clamp protocols shown below traces. The strength of the conditioning step is +30 mV (black trace), 0 mV (grey trace) and −30 mV (blue trace) and the duration is 10 ms in *A* and 100 ms in *B*. *C*, population data showing the decay time constant (tau) of *I*_NaR_ as a function of repolarizing membrane voltage (*V*_MEMBRANE_). *D–F*, population data showing the *I*_NaR_ amplitude as a function of repolarizing membrane voltage (*V*_MEMBRANE_) in response to different conditioning steps. The strength of the conditioning step is +30 mV in *D*, 0 mV in *E* and −30 mV in *F*, and the duration is 10 and 100 ms. Error bar, SEM.

We confirmed the generation of *I*_NaR_ in late‐developing NM neurons (i.e. E19–21), when near‐mature hearing ability is established (Jones *et al*. 2006). Next, we investigated whether NM neurons in the earlier periods of development have *I*_NaR_. Voltage‐clamp experiments were first performed on NM neurons at E14–16, which corresponds to a developmental period when crude hearing ability is just established for chickens (i.e. during hearing onset). To our surprise, we observed robust generation of *I*_NaR_ at E14–16 when using the conditioning step of +30 mV, 10 ms, with the peak amplitude just slightly smaller than that of late‐developing NM neurons (Fig. 8 *A* and *D*). Also similar to late‐developing neurons, the *I*_NaR_ amplitude was reduced when we changed the level of the conditioning step to either less positive voltages (i.e. 0 and −30 mV) or extended the step duration (i.e. 10 and 100 ms) (Fig. 8 *A* and *B* and *D*–*F*). Additionally, we observed a shift in the peak of *I–V* curve in the positive direction by ∼15 mV when we prolonged the duration of the step from 10 to 100 ms. These observations suggest that during hearing onset, the mechanisms of open channel block are established and show near‐mature properties.

**Figure 9:**
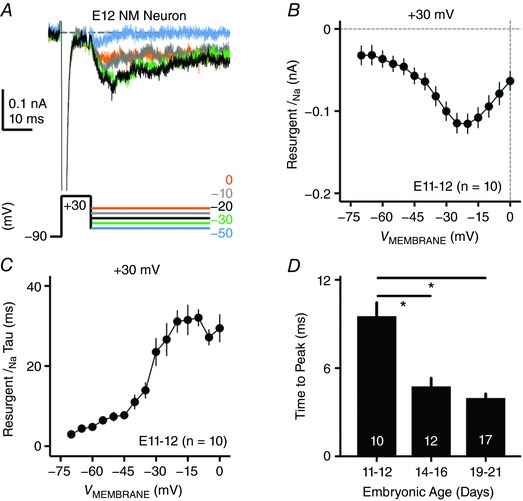
*I*_NaR_ properties of NM neurons at E11–12 *A*, representative current traces in response to voltage‐clamp protocols shown below traces. The conditioning step is +30 mV at 10 ms. *B*, population data showing the current amplitude as a function of repolarizing membrane voltage (*V*_MEMBRANE_) in response to conditioning step of +30 mV at 10 ms. *C*, population data showing the decay time constant (tau) of *I*_NaR_ as a function of repolarizing membrane voltage (*V*_MEMBRANE_). *D*, population data showing time to peak of *I*_NaR_ at different embryonic ages. ^*^ *P* < 0.05. Numbers on the bars represent sample size. Error bar, SEM.

Despite these similarities, we also noticed that some properties of *I*_NaR_ at E14–16 were different from late‐developing neurons. It should be noted that the following differences are discussed using the conditioning step of +30 mV, 10 ms. Although the *I*_NaR_ amplitude peaked at −40 mV for both age groups, it reduced more dramatically at older ages when membrane voltage became more positive or more negative (see Figs 3 *Ac* and 8 *D*). According to a previous study (Lewis & Raman, 2011), the relative amplitude of *I*_NaR_ depends on two mechanisms. First, the rate of the open channel block unbinding from the Na_V_ channel α‐subunit, which indicates the affinity between these two particles, and second, after removal, the rate of the α‐subunit entering the classic inactivation or closed states (depending on the voltage). Time to peak is a good index for the first mechanism (i.e. the rate of unbinding), while decay time constant is dependent on both mechanisms. We did not observe a significant difference of time to peak between two age groups (see Fig. 9 *D*, *P* = 0.88). Furthermore, we plotted the decay time constant as a function of membrane voltage for E14–16 NM neurons and compared it with E19–21 NM neurons (Figs 8 *C* and 4 *B*, respectively). Indeed, E14–16 NM neurons showed a generally larger decay time constant than the older age group. Therefore, we suggest that Na_V_ channels at E14–16 inactivate or close (at the membrane voltage more positive or more negative than −40 mV, respectively) significantly slower after the unbinding of the open channel blocker, resulting in a less dramatic fall‐off of *I*_NaR_ amplitude and thus a shallower slope of their *I–V* curve than Na_V_ channels at E19–21. Although additional experiments are required to test this suggestion, we speculate that longer *I*_NaR_ decay kinetics are partially related to the type of Na_V_ α‐subunit(s) expressed at E14–16 compared to older ages (see below).

Next, we performed voltage‐clamp experiments on NM neurons at E11–12, when chickens are not able to respond to sound (i.e. before hearing onset; Jones *et al*. 2006). In 10 out of 14 recorded neurons, we observed small *I*_NaR_ when using the conditioning step of +30 mV, 10 ms (Fig. 9 *A*, note scale). The maximal amplitude of *I*_NaR_ was slightly above 100 pA, which is dramatically smaller than the current amplitude of the other two age groups (Fig. 9 *B*). In addition, the voltage dependence of *I*_NaR_ at E11–12 was very different from the other two age groups, showing an obvious shift in peak voltage (i.e. at −20 mV, Fig. 9 *B*). Fig. 9 *C* shows the decay time constant of *I*_NaR_ for NM neurons before hearing onset, which presented with an increasing trend with more depolarized membrane voltage. Time to peak was 9.54 ± 2.87 ms when measured at the maximal *I*_NaR_. This value is significantly larger than those of the other two age groups, indicating a stronger affinity between the open channel blocker and the α‐subunit of Na_V_ channels, and thus slower kinetics of *I*_NaR_ at E11–12 (Fig. 9 *D*). Taken together, during the prehearing period, *I*_NaR_ was present in the majority of NM neurons with preliminary properties. With development, the *I*_NaR_ amplitude increased, kinetics improved and its voltage dependence shifted towards negative direction. By the time of hearing onset, NM neurons obtained more mature‐like *I*_NaR_ properties. Table 3 summarizes the developmental changes in amplitude of *I*_NaT_, *I*_NaR_ and *I*_NaP_, when measured at membrane voltage of −30 mV. The amplitude of *I*_NaP_ was measured at the end of 100 ms repolarization after the conditioning step (+30 mV, 10 ms). We observed significant increases in all three current amplitudes for NM neurons as a function of development.

**Table 3:** Sodium channel current properties

Amplitude	E11–12 (*n*)	E14–16 (*n*)	E19–21 (*n*)	ANOVA
Transient *I*_NaV_ a (nA)	−1.57 ± 0.13 (7)	−2.65 ± 0.30 (12)	−3.85 ± 0.40 (12)	*P = *0.0005
Resurgent *I*_NaV_ b (nA)	−0.10 ± 0.01 (10)	−0.44 ± 0.05 (12)	−0.60 ± 0.04 (17)	*P < *0.0001
Persistent *I*_NaV_ b ^,^ c (nA)	−0.05 ± 0.01 (10)	−0.12 ± 0.02 (12)	−0.14 ± 0.01 (16)	*P* = 0.001

*^a^*Transient Na_V_ current = amplitude calculated at step depolarization to −30 mV (holding voltage = −90 mV). *^b^*Resurgent and persistent Na_V_ current = amplitude calculated at the repolarizing membrane voltage of −30 mV. The conditioning step = +30 mV for 10 ms. *^c^*Persistent Na_V_ current = measured at the end of the 100 ms repolarizing membrane voltage.

### Development of frequency–firing pattern in NM

**Figure 10:**
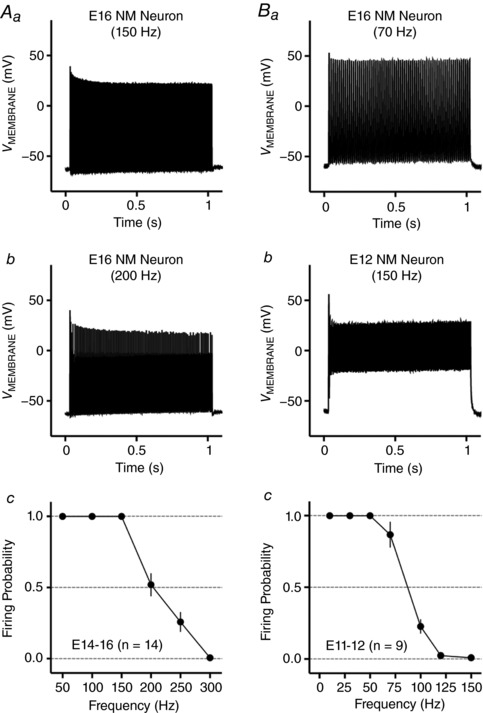
Frequency–firing pattern of NM neurons at E14–16 and E11–12 *Aa* and *b*, representative voltage responses recorded from an E16 NM neuron to current injections of square pulse trains at 150 and 200 Hz, respectively. *Ac*, population data showing firing probability of E14–16 NM neurons as a function of square pulse frequency. The strength of square pulse trains is 1 nA with a duration of 1 s. Individual square pulse width is 2 ms. *Ba* and *b*, representative voltage responses recorded from an E12 NM neuron to current injections of square pulse trains at 70 Hz and 150 Hz, respectively. *Bc*, population data showing firing probability of E11–12 NM neurons as a function of square pulse frequency. The strength of square pulse trains is 1 nA with a duration of 1 s. Individual square pulse width is 5 ms. Error bar, SEM.

Based on the functional role of *I*_NaR_ and its development in NM, we predicted that neurons at the age of E14–16 would show similar frequency–firing pattern with late‐developing neurons (i.e. E19–21), whereas neurons at E11–12 would not be able to follow square pulse frequency as high as the other two age groups, due to their underdeveloped *I*_NaR_ (see Fig. 9) and K_V_ channels (Hong *et al*. 2016). To test this prediction, we first applied the aforementioned current commands of square pulse trains to NM neurons at E14–16. As expected, neurons at this age were able to follow square pulse trains of 150 Hz in a one‐to‐one manner (Fig. 10 *Aa* and *c*). Firing probability dropped continuously when stimulus frequency was higher than 150 Hz (Fig. 10 *Ac*). In response to square pulse trains of 200 and 250 Hz, firing probabilities were ∼0.5 and ∼0.25, respectively (Fig. 10 *Ab* and *Ac*), which are slightly lower than those of E19–21 NM neurons but these differences are not statistically significant (*P* = 0.49 and 0.32, respectively). When the stimulus frequency was 300 Hz, E14–16 NM neurons showed a single onset spike similar to late‐developing neurons (data not shown).

For NM neurons at E11–12, individual square pulse width was extended to 5 ms because neurons at this age are present with average AP half‐width of ∼4.6 ms (Hong *et al*. 2016). We found that NM neurons at E11–12 were able to follow square pulse trains with good fidelity up to 70 Hz (Fig. 10 *Bc*). Fig. 10 *Ba* shows a representative E12 neuron that was able to reliably generate an AP in response to each pulse of the 70 Hz stimulation. When stimulus frequency was increased to 100 Hz or higher, however, firing probability dropped steeply below 0.5 and in response to 150 Hz stimulation, NM neurons at E11–12 only generated a single onset spike followed by membrane oscillations at a depolarized level (Fig. 10 *Bb* and *c*). This is in stark contrast to the other two age groups, which could follow 150 Hz square pulse trains in a reliable manner (see Figs 2 *D* and 10 *Ac*). Taken together, NM neurons at E14–16 showed a frequency–firing pattern closely resembling their late‐developing counterparts, while the ability of NM neurons at E11–12 to follow high‐frequency stimulations was markedly limited. These results are similar to our previous data, which show that E11–12 NM neurons are most responsive to low‐frequency (<40 Hz) sinusoidal current injections (Hong *et al*. 2016).

### Expression of Na_V_1.6 channels

**Figure 11:**
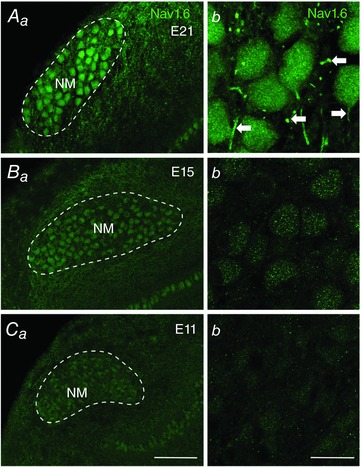
Na_V_1.6 distribution in developing NM Na_V_1.6 immunoreactivity at E21 (*A*), E15 (*B*) and E11 (*C*). Left (*Aa*, *Ba* and *Ca*) and right (*Ab*, *Bb* and *Cb*) columns are low‐ and high‐magnification confocal images, respectively. Dashed lines indicate the boundary of NM. NM, nucleus magnocellularis. Scale bars: 50 μm in *Ca* (left column), 10 μm in *Cb* (right column).

**Figure 12:**
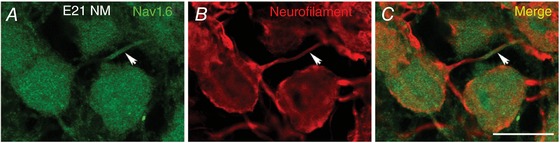
Axonal localization of Na_V_1.6 immunoreactivity at E21 Double staining of Na_V_1.6 and neurofilament in E21 NM. Arrows indicate a distinct segment containing Na_V_1.6, which overlaps with neurofilament‐stained axon that can be traced back to the cell body. NM, nucleus magnocellularis. Scale bars: 20 μm for all panels.

Na_V_1.6 channels are extensively expressed in the mammalian central nervous system and act as a predominant carrier for *I*_NaR_ (Eijkelkamp *et al*. 2012; Lewis & Raman, 2014). Therefore, we explored whether Na_V_1.6 channels are expressed in developing NM, using an antibody specifically recognizing chicken Na_V_1.6 (Kuba *et al*. 2006, 2010, 2014). In late‐developing NM neurons (i.e. E21), strong Na_V_1.6 immunoreactivity was observed as bright punctate segments (Fig. 11 *Aa* and *b*). Because E21 NM neurons are mostly adendritic (Jhaveri & Morest, 1982a), double staining of Na_V_1.6 and neurofilament further demonstrated that Na_V_1.6 is localized in neurofilament‐stained NM axons that can be traced back to the cell bodies (Fig. 12). These Na_V_1.6‐containing segments closely resembled the characterized distribution pattern of Na_V_1.6 in chicken auditory brainstem as reported by Kuba *et al*. (2010, 2014) and possibly represented the axon initial segments (AIS) and nodes of Ranvier. In contrast, the Na_V_1.6‐containing segments were absent in NM at E15 (Fig. 11 *Ba* and *b*) and E11 (Fig. 11 *Ca* and *b*). In addition, NM cell bodies contained a low level of Na_V_1.6 immunoreactivity at E21, which became increasingly less distinct from E21 to E15 and from E15 to E11.

## Discussion

Auditory brainstem neurons of vertebrates fire phase‐locked APs at high rates with remarkable fidelity. We show here that late‐developing NM neurons fire reliable APs to square‐pulse current injections up to 200 Hz. In addition to the recognized role of K_HVA_ channels in this process, we also report that *I*_NaR_ of Na_V_ channels is an equally important component that operates synergistically with K_HVA_ channels to enable rapid AP firing in NM, an evolutionarily conserved process between birds and mammals likely promoting similar hearing functions. To our surprise, small *I*_NaR_ responses were present during a prehearing period (E11–12). At hearing onset (E14–16), *I*_NaR_ properties closely resemble late‐developing NM neurons (E19–21), despite developmental refinement in Na_V_ channel protein expression patterns. In line with these results, NM neurons at E14–16 showed comparable frequency–firing ability to their late‐developing counterparts, whereas NM neurons at E11–12 are most responsive to lower‐frequency stimulations.

### Factors regulating AP firing patterns in NM

K_HVA_ channels are a common regulator of AP kinetics (Rudy & McBain, 2001; Johnston *et al*. 2010). In the auditory brainstem of both birds and mammals, K_V_3‐containing channels are abundantly expressed (Wang *et al*. 1998; Parameshwaran *et al*. 2001; Parameshwaran‐Iyer *et al*. 2003). Blockade or knockout of K_V_3 channels results in wider APs that undermine the ability to generate APs at high rates (Wang *et al*. 1998; Klug & Trussell, 2006). We report a similar result here (Fig. 2). In addition, a recent study on *Xenopus* oocytes shows that K_V_3.1 subunits are able to produce resurgent potassium current during repolarization, which provides an additional repolarizing drive to the membrane (Labro *et al*. 2015). This property is not due to the open channel blocker but to the unique gating kinetics of K_V_3.1 subunits, and it facilitates the termination of APs and thus likely promotes high‐frequency repetitive firing. Nevertheless, it should be noted that 1 mm TEA used in the current study can also block a small portion of K_V_7‐containing channels and calcium‐activated BK channels (Johnston *et al*. 2010). K_V_7‐containing channels are one of the K_LVA_ channels with slow activation kinetics (Johnston *et al*. 2010). They contribute minimally in NM and their immunoreactivity is minimal compared to K_V_3 and K_V_1 (Kuba *et al*. 2015). Due to their weak expression, blockade of K_V_7‐containing channels in NM neurons does not induce significant changes in AP properties (Kuba *et al*. 2015). In contrast, BK channels play an important role in regulating AP kinetics in many other neurons (Kimm *et al*. 2015). However, a previous study in NM reported no change in K_V_ current when external calcium was replaced by cobalt ions, suggesting minimal contribution of calcium‐activated potassium current to the total current (Koyano *et al*. 1996). Therefore, K_V_3‐containing channels are probably the primary targets of 1 mm TEA applied in our experiments.

Another factor that regulates AP firing pattern is fast inactivation kinetics of Na_V_ channels (i.e. the steep decay slope of *I*_NaT_) observed in auditory brainstem neurons (Ming & Wang, 2003; Hong *et al*. 2016). As demonstrated by double‐pulse experiments, a second *I*_NaT_ can fully recover within 5 ms following the initial pulse (Leao *et al*. 2005; unpublished observation in NM), which explains in the current study why NM neurons are able to reliably follow the stimulus frequency at 150 Hz (interspike interval = 6.7 ms, Fig. 2 *D*) after blockade of K_HVA_ channels. Finally, K_LVA_ channels with fast activation kinetics, such as K_V_1‐containing channels, work as another regulator of AP firing by controlling the time constant of passive membrane properties, shaping the speed of membrane voltage changes (Klug & Trussell, 2006).

*I*_NaR_ is an additional factor in regulating AP firing rates of NM neurons. The open channel block promotes Na_V_ channel availability and recovery immediately after brief depolarization in NM, a result consistent with previous studies (Raman & Bean, 2001; Patel *et al*. 2015). However, we report two major differences. First, Na_V_ channel availability is much higher in our study at any given recovery time period (Fig. 5 *C*). This is likely because our recordings were made from intact neurons in brainstem slices that have significantly more neuron‐specific Na_V_ channels. Previously reported data were obtained from the soma of dissociated Purkinje cells or from HEK cells transfected with Na_V_ channels. Second, the distinction in recovery time constant between the open channel block and classic inactivation state is smaller in our study (∼1 ms *versus* > 5 ms, Fig. 5 *D*). This may be attributed to relatively fast kinetics of *I*_NaT_ in auditory brainstem neurons. However, the extent to which the open channel block or the classic inactivation gate contributes to rapid *I*_NaT_ kinetics in NM is unclear.

‘Real‐time’ *I*_NaR_ appears to be a relatively small inward Na_V_ current during repolarization from an initial *I*_NaT_, as shown by our model NM neuron (Fig. 7 *Ad*). This observation closely resembles experimental data when AP waveforms are used as voltage commands and the dynamic changes in Na_V_ current are documented (i.e. AP‐clamp method; Raman & Bean, 1997, 1999). As seen from studies that utilized this technique, the *I*_NaR_ provided a small depolarizing drive immediately after an AP, important for subsequent AP firing (Raman & Bean, 1997). However, AP‐clamp is susceptible to various technical limitations (e.g. space clamp errors), and as a result the majority of these studies were conducted on dissociated neurons (Raman & Bean, 1997; Do & Bean, 2003). Nevertheless, there are successful slice recordings with AP clamp from adendritic mesencephalic trigeminal neurons (Enomoto *et al*. 2006) Given the fact that the majority of late‐developing NM neurons are also adendritic, AP‐clamp experiments could provide valuable insight into the biological relevance of *I*_NaR_. There are still technical limitations (e.g. sample rate, kinetics, immature dendritic NM neurons, etc.) and therefore we employed a computational model to better address its functional significance. Indeed, removal of *I*_NaR_ reduced AP firing rate at 200 Hz for our model NM neuron (Fig. 7). Yet, the effects on Na_V_ channels after the knockdown of open channel blocker remain to be determined, especially regarding the kinetics of *I*_NaT_ under experimental condition. These effects are dependent on the types of β‐subunits expressed in NM neurons and their specific interactions with Na_V_ α‐subunits (Qu *et al*. 2001; Aman *et al*. 2009; Bant & Raman, 2010). Therefore, our computational model of NM neuron is subject to future improvements when more information about molecular substrates for the open channel blocker in NM is obtained.

### Development of resurgent Na_V_ current in NM

The inner ear of chickens only responds to loud sound (>80 dB) and afferent ganglion neurons present with poor frequency selectivity at E14–16 (Jones *et al*. 2006). It is surprising at this age that *I*_NaR_ properties are relatively established, except for decay kinetics, which are due in part to underdeveloped Na_V_ α‐subunit (Fig. 8, see below). *I*_NaR_ properties at E11–12 – a developmental period when the auditory system does not respond to sound and is considered ‘prehearing’ – differ greatly from those of the other age groups (Fig. 9). *I*_NaR_ development in NM parallels the development of frequency–firing pattern (Fig. 10), along with maturation of intrinsic AP properties and K_V_ channel conductances as we previously reported (Hong *et al*. 2016). NM neurons at E14–16 generate APs as fast and reliably as late‐developing neurons. Additionally, properties of K_LVA_ and K_HVA_ currents are underdeveloped at E11–12 but become more mature‐like around hearing onset. Taken together, NM neurons have near‐mature ion channel properties around hearing onset, suggesting a priming period during hearing development for establishing mature auditory functions.

*I*_NaR_ at E11–12, though small, may be important for shaping NM's AP firing pattern. In response to low‐frequency (5–10 Hz) sinusoidal current injections, NM neurons at this age generate short bursts of APs per cycle, despite the fact that their overall low K_V_ channel conductances are not sufficient to work against the largely depolarized membrane voltage during bursts (Hong *et al*. 2016). *I*_NaR_ also contributes to burst firing in other non‐auditory neurons (Enomoto *et al*. 2006, 2007). We speculate that *I*_NaR_ at E11–12, albeit reduced, helps generate burst firing for immature NM neurons, but its functional significance at this age is unclear.

Anatomically, we detected strong Na_V_1.6 expression at a late‐developing stage (E21), which was dramatically reduced at hearing onset (E14–16) and prehearing (E11–12) periods (Fig. 11). Additionally, the majority of Na_V_1.6 channels in late‐developing neurons are located along the axons, while this distribution pattern is not evident for the other age groups. The Na_V_1.6‐positive punctate segments in late‐developing NM neurons are likely to be the AIS and the nodes of Ranvier, respectively, though future immunocytochemistry with double staining of ankyrin G, a scaffold protein that marks the AIS, is needed to confirm this speculation (Kuba *et al*. 2014). The presence of *I*_NaR_ at E11–16 suggests that additional Na_V_ subtypes may contribute to *I*_NaR_ during development of NM neurons. The following evidence supports this suggestion. First, strong expression of Na_V_1.2 channels is observed at E11–15 in NM's postsynaptic target nuclei (i.e. nucleus laminaris) (Kuba *et al*. 2014). Second, studies in spinal sensory neurons report the generation of *I*_NaR_ from different Na_V_ subtypes, including Na_V_1.2 channels (Rush *et al*. 2005; Jarecki *et al*. 2010).

### Resurgent Na_V_ current is conserved across species and structures

*I*_NaR_ has been reported as a conserved property in numerous neuronal types in the mammalian cerebellum, cortex, brainstem and spinal cord (Afshari *et al*. 2004; Cummins *et al*. 2009; Lewis & Raman, 2014). As for the mammalian auditory system, *I*_NaR_ has been found at the calyx of Held and its postsynaptic target – the medial nucleus of trapezoid body (Leao *et al*. 2005; Kim *et al*. 2010). The only report of *I*_NaR_ beyond the scope of mammals comes from chicken Purkinje cells (Lewis & Raman, 2011). Our results from the current study further confirm its contribution in the chicken auditory brainstem. Here, the AP firing pattern of NM neurons (and the analogous bushy cells of the mammalian anteroventral cochlear nucleus) is essential for the encoding of behaviourally relevant acoustic cues. Central to this are established and specialized structural and functional features shared across species (Carr & Soares, 2002; Koppl, 2009; Grothe *et al*. 2010). The report of *I*_NaR_ in the current study strongly suggests yet another physiological feature shared across species within the central nervous system.

Despite the distribution of *I*_NaR_ in the central nervous system (Lewis & Raman, 2014), its underlying molecular substrates are unclear. The Na_V_ β4‐subunit has been identified as one important open channel blocker in cerebellar Purkinje cells, granule cells and dorsal root ganglia neurons (Grieco *et al*. 2005; Bant & Raman, 2010; Barbosa *et al*. 2015). Interestingly, the amino acid sequence of the β4‐subunit is also conserved across species, from frogs to mammals, raising the possibility of its being the open channel blocker for avian NM neurons (Lewis & Raman, 2011). Nevertheless, questions are raised of whether the β4‐subunit is the only possible open channel blocker. For example, both perirhinal and entorhinal pyramidal neurons show robust *I*_NaR_ but not β4 expression at high levels, possibly indicating multiple molecular substrates of *I*_NaR_ (Castelli *et al*. 2007; Nigro *et al*. 2012). In addition, FGF14, an intracellular protein and a member of the fibroblast growth factor homologous factors (FHFs), has been proposed as another key player in open channel block in Purkinje cells (Yan *et al*. 2014). A recent study on dorsal root ganglia neurons demonstrated that another member of the FHF family, FHF2, plays an important regulatory role for *I*_NaR_ (Barbosa *et al*. 2017). Interestingly, two isoforms of FHF2 have opposite effects on the amplitude of *I*_NaR_. As for NM neurons, it should be noted that differences in *I*_NaR_ across development, especially at E11–12, might be attributed not only to different expression of Na_V_ α‐subunits, but also to changes in the open channel blocker molecular substrates themselves. Therefore, what the underlying open channel blockers for NM neurons are and how the developmental expression of these blockers shapes *I*_NaR_ maturation are interesting questions for future studies.

## Additional information

### Competing interests

The authors declare no competing financial interests.

### Author contributions

All authors have approved the final version of the manuscript and agree to be accountable for all aspects of the work. All persons designated as authors qualify for authorship, and all those who qualify for authorship are listed. HH, TL, XW, YW and JTS designed the study. HH performed electrophysiology experiments at Northwestern University. TL performed computational modeling experiments at Northwestern University. XW and YW performed immunocytochemical experiments at Florida State University. HH, TL, XW, YW and JTS analyzed/interpreted data and wrote the manuscript.

### Funding

This research was supported by the National Institute on Deafness and Other Communication Disorders (NIDCD) DC013841 (J.T.S.) and the Hugh Knowles Hearing Research Center (J.T.S.).
